# The impact of gambling advertising on gambling severity: a path analysis of factors of psychological distress in individuals with gambling disorder

**DOI:** 10.3389/fpsyg.2025.1523906

**Published:** 2025-03-14

**Authors:** Hibai Lopez-Gonzalez, Roser Granero, Fernando Fernández-Aranda, Mark D. Griffiths, Susana Jiménez-Murcia

**Affiliations:** ^1^Department of Library, Information Science and Communication, University of Barcelona, Barcelona, Spain; ^2^Department of Psychobiology and Methodology, Autonomous University of Barcelona, Barcelona, Spain; ^3^Psychoneurobiology of Eating and Addictive Behaviors Group, Neurosciences Programme, Bellvitge Biomedical Research Institute (IDIBELL), Barcelona, Spain; ^4^Ciber Physiopathology of Obesity and Nutrition (CIBERObn), Instituto de Salud Carlos III, Barcelona, Spain; ^5^Clinical Psychology Department, Bellvitge University Hospital-Bellvitge Biomedical Research Institute (IDIBELL), Barcelona, Spain; ^6^Department of Clinical Sciences, School of Medicine and Health Sciences, University of Barcelona, Barcelona, Spain; ^7^International Research Gambling Unit, Psychology Department, Nottingham Trent University, Nottingham, United Kingdom

**Keywords:** gambling advertising, gambling disorder, path analysis, clinical setting, impulsivity, emotion regulation

## Abstract

**Background:**

There is a consensus in the literature that gambling advertising disproportionately affects those experiencing higher gambling severity. However, the relationship of gambling advertising and severity is typically assessed among samples recruited from online panels using screening tools as the method to categorize the gambling severity status of participants. Alternatively, other studies use small groups of gamblers (i.e., qualitative studies). The present paper reports findings from a sample of gamblers diagnosed with gambling disorder by professional clinicians via individual interviews. The study investigated the association between gambling advertising and gambling severity by looking at other psychologically relevant variables such as impulsivity, emotion regulation, and general psychopathology.

**Methods:**

A sample of 210 consecutive treatment-seeking patients was recruited from a public hospital from June 2019 to January 2021. A path analysis model was run to determine the relationship between the variables. Gambling advertising was constructed as a latent variable, comprising the perceived impact of gambling advertising, persuasion knowledge, and the attitudes towards gambling advertising.

**Results:**

Gamblers with greater gambling severity reported higher perceived impact of gambling advertising, and more positive attitudes toward gambling advertising. Gambling advertising was a mediator in the paths between emotion regulation and gambling severity, and between impulsivity and gambling severity.

**Conclusion:**

The study demonstrates among individuals with verified gambling disorder that there is a relationship between gambling advertising and gambling severity. Regulators have an empirical basis on which to restrict the exposure to gambling advertising of vulnerable groups.

## Background

Marketing and advertising strategies intend to make gambling products more attractive to consumers and are generally considered a situational factor that can play a part in the development of gambling-related harm (Griffiths, 2005; Browne et al., 2019). Gambling advertising emphasizes specific aspects of gambling including how easy it is to win, how glamorous a gambler’s life is, how normal and frequent is to gamble, and how easy it is to use gambling apps (McMullan and Miller, 2008; Sklar and Derevensky, 2011; Milner and Nuske, 2012; Lopez-Gonzalez et al., 2018). As a product designed with a negative expected financial return for gamblers, it is essential for its viability to deliver commercially biased information (i.e., advertising) to persuade consumers to think that gambling is a skill-based game that can be mastered (Toneatto et al., 1997).

There is a growing consensus in western societies that the amount of gambling advertising the population is regularly exposed to is excessive, and that measures should be taken in order to reduce it (Hing et al., 2015b; Hörnle and Carran, 2018; Sharman, 2022). Recently, countries such as the UK, Spain, Norway, the Netherlands, Italy, Belgium, and Australia have passed laws that either restrict or outright ban multiple forms of gambling advertising (McLoughlin and Heery, 2022). These changes in gambling advertising regulation have prompted operators to look for alternative ways to reach their targeted audience. The mounting pressure that gambling regulators are imposing on what gambling advertisers can do is incentivizing a transformation in the gambling advertising market. Traditional television and radio advertisements are being substituted by social media marketing strategies in which limiting the exposure to vulnerable groups becomes harder (Guillou-Landreat et al., 2021). A mixed-methods study using big data from *Twitter* and a manual codification technique found that gambling advertisements on social media are often unclear and inexplicit about their promotional nature, and that the normalization effect they produce can be larger than in traditional advertising because of the sheer volume of inducements individuals are exposed to (Rossi et al., 2021).

Gambling advertising restrictions are based on the assumption that they can cause harm to vulnerable individuals (e.g., individuals with gambling problems and adolescents) (McGrane et al., 2023). However, establishing the real impact of gambling advertising and understanding its mechanisms of influence has been an aim of gambling research for some time (Binde, 2014). One of the most intuitive approaches to establishing the effects of gambling advertising on gambling behaviour concerns total consumption theory. In simple terms, if the number of individuals experiencing gambling-related harm varies as a fixed rate of the total number of individuals engaging in gambling activities, then gambling advertising increases gambling harm simply because it increases the overall number engaging in gambling behaviour at population level (Lund, 2008; Hansen and Rossow, 2012; Hing et al., 2014). A similarly intuitive approach concerns paying attention to how much money the gambling industry spends on advertising. It was reported that gambling advertisements ranked 11^th^ among 1,200+ product categories advertised on US television (Nielsen, 2021). Another study calculated that from January 2019 to July 2021, gambling operators spent £972 million on advertising in the British market (Critchlow et al., 2023). Arguably, if gambling advertising had no effect whatsoever, gambling companies would not spend their money on it. Nonetheless, none of these methods clearly demonstrate the detrimental impact of gambling advertising.

If gambling advertising is effective, one of the most concerning consequences of its effectiveness would be its impact on the most vulnerable groups. There appears to be a consensus that the proportion of gambling disorder (GD) solely attributable to the effects of advertising, if any, must be low (Binde, 2014), with other individual factors (e.g., biological and psychological characteristics) along with situational and structural factors (e.g., regulation, availability, speed of play) understood to be more significant determinants of GD (Grant et al., 2010; Slutske, 2019).

However, many studies repeatedly suggest that gambling advertising disproportionally affects those already suffering gambling problems. In an online panel with 1,000 Australian adults, researchers found that sports bettors experiencing gambling problems reported that sports-embedded gambling promotions maintained or worsened their gambling behaviour. Betting on sports in the next 6 months was predicted by being exposed to gambling promotions, previous sports betting participation, and higher problem gambling severity (Hing et al., 2015a). In another sample with Australian college students, those who reported more positive attitudes towards gambling advertising and more frequent sport watching also scored higher on a problem gambling screening instrument (Hing et al., 2013). In a sample of 1,148 adolescents from Quebec, Canada, it was found that those with gambling problems also reported higher recall of and exposure to gambling advertising, and that they were more vulnerable to suggestions to keep playing and that a big win was imminent (Derevensky et al., 2010). A study with 131 people with GD found that 46% reported that gambling advertising triggered them to gamble (Grant and Kim, 2001). In a national survey in Norway, those scoring higher on a problem gambling screen also reported higher exposure to gambling advertising (Hanss et al., 2015). When asked, participants agreed that advertising increased their involvement with gambling, although in general, most gamblers only acknowledged that gambling advertising made them more aware of different gambling brands available in the market, but that it did not influence their attitudes, interest, and behaviour. In a more recent Norwegian study with general population, exposure to direct gambling (receiving texts, emails, telephone calls) was linearly associated with gambling severity (Syvertsen et al., 2022). Other studies have reported similar evidence concerning gambling advertising and gambling problems (Gavriel Fried et al., 2010; Clemens et al., 2017). Overall, there is much evidence indicating an association between gambling severity and perceived/self-reported impact of gambling advertising, although the causality of such association remains unknown (McGrane et al., 2023).

Correspondingly, there is also empirical evidence from qualitative studies interviewing gamblers about the impact they perceive gambling advertising had on their behaviour. In two interview studies from Sweden and Spain, individuals experiencing gambling problems acknowledged that advertising was a source of distress for them, but rarely identified it as the main cause of their gambling problems (Binde, 2009; Lopez-Gonzalez et al., 2020b). Other interview studies with Australian participants have also explored the perceptions of gamblers with gambling problems about their difficulties to resist gambling enticements (Thomas et al., 2012; Deans et al., 2017).

To better understand the association between gambling severity and advertising, the present study also examined other relevant psychological dimensions that might help to explain it. In particular, emotion regulation, impulsivity, personality, and general psychopathology were examined. Emotion regulation (ER) is the process of modifying the intensity and/or duration of the emotions an individual experiences as a result of contextual demands (Mestre-Bach et al., 2020). Numerous studies have shown that individuals with GD are at higher risk of presenting emotion dysregulation than those without GD (Williams et al., 2012; Estévez et al., 2017; Rogier et al., 2020). In the case of gambling advertising, advertisements could entice gamblers into trying to regulate their emotional states through gambling. Moreover, some forms of gambling such as sports betting are of significant emotionality as they include sports and athletes that often symbolically represent the gamblers’ own territorial and personal identities and affiliations.

Impulsivity is another well-studied psychological factor that interacts with GD, with some of its dimensions such as sensation-seeking and negative urgency showing capacity to predict the GD severity (Nower and Blaszczynski, 2006; Schreiber et al., 2012; Savvidou et al., 2017; Ioannidis et al., 2019). The short-term effects of advertising are also intimately related to impulsiveness because they seek to generate an emotional and instant response on the consumer (Chopdar et al., 2022). Based on the Likelihood Elaboration Model (Petty and Cacioppo, 1986), if advertisements are designed in a way that they provoke gamblers to process messages via the peripheral route (i.e., more emotionally, via affective associations) rather than the central route of processing (i.e., thoughtful consideration of arguments), they will arguably elicit more impulsive responses. Newer forms of gambling include instantaneous gambling products such as microbets (Russell et al., 2018), and in-play betting (Lopez-Gonzalez et al., 2020a), which markedly shorten the time available for gamblers to make an informed purchase decision.

Individuals with GD often show distinctive patterns in terms of their personality traits (Mestre-Bach et al., 2022). Impulsivity, lack of perseverance, and suspiciousness have been associated to GD by means of emotion regulation (Rogier et al., 2020). Recreational gamblers show less neuroticism than those experiencing GD (Whiting et al., 2019), and an earlier onset of GD has also been associated with a higher degree of novelty-seeking and self-directedness (Odlaug et al., 2013). Therefore, personality could also be involved in the relationship between gambling advertising and gambling severity.

Most of the aforementioned studies did not use quantitative methods with clinically diagnosed samples to understand the impact of gambling advertising on GD (except Grant and Kim, 2001). To date, the published research either (i) examines small samples of clinically diagnosed gamblers with qualitative methods such as focus groups and individual interviews, or (ii) examines larger samples with quantitative methods, but infers GD through self-administered screening tools such as the *Problem Gambling Severity Index* (Ferris and Wynne, 2001), or other behaviour-tracking instruments. These samples are seldom gathered using nationally representative samples, and most often use online panels, in which problem gambling systematically appears to be overestimated when compared to in-person interviews (Sturgis and Kuha, 2022). These singularities impose inescapable limitations about the robustness and generalizability of their results.

Therefore, the present study departs from such approaches by assembling a relatively large sample of gamblers with a GD diagnosis confirmed by clinical specialists using both psychometrically-validated diagnostic instruments and in-person interviews. As aforementioned, only one prior study (Grant and Kim, 2001) has explored gambling advertising effects using a relatively large sample of gamblers from a clinical setting with a certified GD diagnosis issued by professional clinicians, but it was carried out more than two decades ago. The aim of the present study was to examine the association between gambling severity and the impact of gambling advertising, considering how other relevant clinical determinants (e.g., impulsivity, personality, general psychopathology, and emotion regulation) might influence such association. Consequently, a path analysis model was designed to explore the different studied variables in a comprehensive manner.

## Method

### Participants and procedure

The sample comprised consecutive treatment-seeking adults with a diagnosed GD (*n* = 210). The recruitment was carried out from June 2019 to January 2021 in the Behavioural Addictions Unit of a large public hospital in Spain covering a populated area of over 2 million inhabitants in the greater Barcelona area. The GD diagnosis was assessed using DSM-5 criteria (American Psychiatric Association, 2013), SOGS criteria (Lesieur and Blume, 1987), and confirmed in an in-person interview by clinical psychologists and psychiatrists. Inclusion criteria for participation in the study were (i) being an adult, (ii) signing the consent form, (iii) a GD diagnosis, and (iv) GD being their primary reason to seek treatment. No exclusion criteria were predetermined.

All the participants completed a paper-and-pencil self-administered survey before their 16-week cognitive-behavioural therapy for GD started. No individual who met the criteria to join the study refused to participate, although two forgot to sign the consent form and were excluded from the study. In total, 218 questionnaires were completed. Eight were discarded for various reasons (e.g., responding to every single item identically, not signing the consent form), resulting in a final sample of 210 participants. A previous study using the same sample (Lopez-Gonzalez et al., 2024) was published before but it differed in the statistical analyses performed and the consideration of psychological distress, emotion regulation, and impulsivity as variables of interest.

### Measures

Impacts of Gambling Advertising Scale (Hanss et al., 2015) (HANSS). The nine-item instrument based on a previous scale (Derevensky et al., 2010) was used to assess the perceived impact of gambling advertising on three subscales: involvement (e.g., *“I am more likely to gamble after seeing a gambling advertisement”*), awareness (e.g., *“I do not pay attention to gambling advertisements”*), and knowledge (e.g., *“Gambling advertisement has increased my knowledge of gambling providers”*). A Spanish adaptation was conducted using a translation and back-translation technique. Participants rate the nine items on a four-point scale (from *“strongly agree”* to *“strongly disagree”*). Lower scores indicate a greater perceived impact of gambling advertising. The original scale had Cronbach’s alpha values of 0.84 (involvement), 0.64 (awareness), and 0.85 (knowledge). In the present study, internal consistency was very good (α = 0.86).

Consumer Sentiment Toward Marketing (Gaski and Etzel, 1986) (GASKI). The instrument comprises multiple subscales, but only the ‘Advertising for Products’ was used in the present study. This subscale comprises seven items on a five-point rating scale (1 = *“Agree strongly,”* 5 = *“Disagree strongly”*) that assess attitudes towards advertising. For the Spanish adaptation, the subscale was slightly modified to represent gambling advertising rather than general advertising. A translation and backtranslation was carried out. The original “Advertising for products” scale obtained very good reliability (α = 0.76). In the present study, internal consistency was even greater (α = 0.80).

Consumer Self-Confidence on Persuasion Knowledge (Bearden et al., 2001) (BEARDEN). The six-item instrument was used to assess how confident individuals are in identifying the tactics of marketers to persuade them to buy products. Items (e.g., *“I know when an offer is too good to be true”*) are rated from 1 = *“extremely uncharacteristic”* to 5 = *“extremely characteristic.”* The scale was adapted to Spanish using a translation and backtranslation technique. The original scale had a Cronbach’s alpha of 0.83. In the present study, internal consistency was excellent (α = 0.91).

South Oaks Gambling Screen (Lesieur and Blume, 1987) (SOGS). Spanish version (Echeburúa et al., 1994). The 20-item SOGS was used to assess gambling problems. The responses categorize individuals into three groups: non-problem, probable pathological, and problem gamblers. This study used the Spanish validation of the scale, which achieved very good psychometrical results (test–retest reliability R = 0.98, internal consistency α = 0.94 and convergent validity R = 0.92). In the present study, internal consistency was good (α = 0.75).

Difficulties in Emotion Regulation Scale (Gratz and Roemer, 2004; Gómez-Simón et al., 2014). The 36-item DERS was used to assess emotional dysregulation. It comprises six subscales: (i) lack of emotional awareness (difficulties in attending to and acknowledging emotions), (ii) lack of emotional clarity (extent to which individuals know and are clear about their emotions), (iii) non-acceptance of emotional responses (tendency to have negative secondary emotional responses to one’s negative emotional states), (iv) difficulties engaging in goal-directed behaviour (difficulties in concentrating and accomplishing tasks when experiencing negative emotions) (v) limited access to emotion regulation strategies (belief that individuals can do little to effectively regulate emotions when being upset) and (vi) impulse control difficulties (difficulties in remaining in control of one’s behaviour when experiencing negative emotions). The previously reported psychometric characteristics of the instrument were excellent (Cronbach’s alpha of 0.93; range = 0.73–0.91). In the present study, the score calculated for the total scale was considered as a measure of the global difficulties with the emotion regulation, and it had excellent internal consistency (α = 0.93).

Impulsive Behaviour Scale (Whiteside et al., 2005). Spanish version (UPPS-P). The 59-item UPPS-P was used to assess five impulsivity traits: (i) lack of perseverance, (ii) lack of premeditation, (iii) sensation seeking, (iv) negative urgency, and (v) positive urgency. Cronbach’s alpha coefficients for the Spanish version of UPPS-P ranged from 0.61 to 0.81. In the present study, the total score was used as a measure of the global impulsivity level, and the internal consistency was very good (α = 0.80).

Symptom Checklist-Revised (SCL-90-R) (Derogatis, 1994) Spanish version (Derogatis, 1997). The 90-item SCL-90-R assesses nine dimensions of general psychopathology (somatization, obsessive-compulsive, interpersonal sensitivity, depression, anxiety, hostility, phobic anxiety, paranoid ideation, and psychoticism), and three global indices (global severity index [GSI], positive symptom total [PST], and positive symptom distress index [PSDI]). Cronbach’s alpha coefficients for SCL-90-R in the Spanish validation ranged from 0.77 to 0.90. In the present study, the SCL-90R PST score was used as measure of the global psychopathology distress, and the internal consistency was excellent (α = 0.98).

Sociodemographic and other gambling-related variables. Sociodemographic data were collected by means of individual in-person interviews with a clinical psychologist including age, gender, employment status, marital status, educational attainment, and social index (Hollingshead, 2011). Additional gambling-related data were collected including onset and duration of GD, gambling modality (in person, online, and mixed), gambling type (strategic, non-strategic, and mixed), engaging in illegal acts to fund gambling behaviour, and indebtedness.

### Statistical analysis

The statistical analysis was carried out with Stata18 for Windows (StataCorp, 2023). Path analysis was implemented using structural equation modeling (SEM). This procedure is a straightforward extension of multiple regression and factor analysis, with different applications. Path analysis has been historically used to disprove a model that postulates potential ‘causal’ relations between the variables, but in the last two decades studies suggest that it can be used for both confirmatory and exploratory modeling, and therefore for theory testing and theory development (MacCallum and Austin, 2000). In this work, path analysis was used as an exploratory method, and the model specification (rationale for the path-diagram) was based on the cumulative empirical evidence presented in the introductory section. Due to the multiple measures for the model, a latent variable was defined by the observed indicators for assessing the advertising profile. The latent variable in the study allowed a simplifying of the data structure and therefore facilitated a more parsimonious fitting (Borsboom et al., 2003). All parameters were free-estimated (they assumed any value and estimated by the SEM) and the maximum-likelihood estimation (MLE) method of parameter estimation was used. Goodness-of-fit was evaluated using standard statistical measures: chi-square test (*χ*^2^), the root mean square error of approximation (RMSEA), Bentler’s Comparative Fit Index (CFI), the Tucker-Lewis Index (TLI), and the standardized root mean square residual (SRMR). The following criteria were considered for adequate model fit (Barrett, 2007): non-significant by the *χ*^2^ test, RMSEA<0.08, TLI > 0.90, CFI > 0.90, and SRMR<0.10. Regarding the adequacy of the sample size for the SEM, it must be considered that some studies have analyzed (through Monte-Carlo procedures) requirements for SEMs, including variation by the number of factors, number of indicators, strength of the indicator loadings and the regressive paths and the amount of missing data per indicator. Focused on the statistical power, bias in the parameter estimates and overall solution propriety, results revealed that the sample requirements were into a very broad range (from 30 to 460, depending on the analysis characteristics), and that solutions that met fitting at a given sample size tended to be stable relative to the results of the analysis at the next largest sample sizes (Von Oertzen, 2010; Wolf et al., 2013). Also, the use of MLE has provided consistent coefficients with relatively large sample sizes (a generally accepted minimum is 200 observations) (Kline, 2023).

### Ethics

The research was conducted in accordance with the Declaration of Helsinki of 1975 (revised 2000), and approved by the Ethics Committee of Bellvitge University Hospital (Ref: PR338/17-CSI 18/04). All participants provided informed consent and received no monetary compensation for participating.

## Results

### General descriptors

Supplementary Table S1 displays the distribution for all the study variables analyzed. Most participants were male (92.9%), single (53.8%), with primary education level (45.2%) and had mean-low to low socio-economic positions (77.2%). Mean age was 39.4 years (*SD* = 13.3). The mean age of onset of gambling-related problems was 28.9 years (*SD* = 11.9) and the mean duration of the problems was 5.2 years (*SD* = 5.7).

### Path analysis

**Figure 1:**
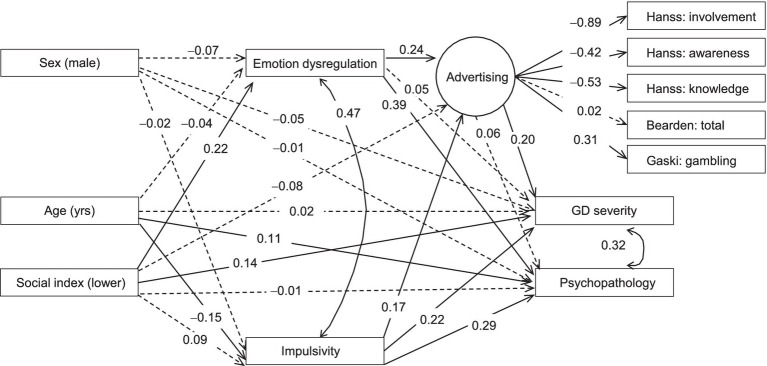
Path diagram: standardized coefficients. Continuous line: significant parameter. Dash line: non-significant parameter. Emotion dysregulation measured as the DERS total. Impulsivity measured as the UPPS-P total. GD severity measured as the SOGS total. Psychopathology measured as the SCL-90R PST. Sample size: *n* = 210.

Figure 1 shows the path diagram, with the standardized coefficients. Adequate goodness of fit was obtained: *χ*^2^ = 43.31 (*p* = 0.088); RMSEA = 0.041 (95%CI: 0.001 to 0.070); CFI = 0.969; TLI = 0.939; SRMR = 0.050.

Table 1 contains the complete results obtained in the SEM (test for direct, indirect, and total effects).

**Table 1:** Results obtained in the SEM: test for direct, indirect and total effects.

		*B*	*SE*	*z-stat*	*p*	*Std-B*
Direct effects: structural						
Impulsivity	Age	−0.2688	0.1220	−2.20	0.028	−0.1506
	Sex (male)	−1.6326	6.2950	−0.26	0.795	−0.0177
	Social index (lower)	1.9968	1.5689	1.27	0.203	0.0866
GD severity	Emotion dysregulation	0.0112	0.0109	1.03	0.304	0.0804
	Impulsivity	0.0308	0.0103	3.00	0.003	0.2235
	Advertising	1.0000	(constrd)			0.2012
	Age	0.0053	0.0159	0.34	0.737	0.0217
	Sex (male)	−0.6154	0.8104	−0.76	0.448	−0.0486
	Social index (lower)	−0.4370	0.2090	−2.09	0.037	−0.1376
Psychopathology	Emotion dysregulation	0.3621	0.0609	5.94	0	0.3887
	Impulsivity	0.2697	0.0586	4.60	0	0.2923
	Advertising	2.1435	1.7837	1.20	0.229	0.0644
	Age	0.1822	0.0919	1.98	0.048	0.1106
	Sex (male)	−0.5200	4.6944	−0.11	0.912	−0.0061
	Social index (lower)	−0.2850	1.2013	−0.24	0.812	−0.0134
Advertising	Emotion dysregulation	0.0067	0.0043	1.57	0.117	0.2388
	Impulsivity	0.0046	0.0033	1.42	0.156	0.1666
	Social index (lower)	−0.0384	0.0525	−0.73	0.465	−0.0600
Emotion dysregulation	Age	−0.0666	0.1190	−0.56	0.576	−0.0376
	Sex (male)	−6.2096	6.1437	−1.01	0.312	−0.0682
	Social index (lower)	5.1201	1.5312	3.34	0.001	0.2244
Direct effects: measurement						
	Involvement	−1.4184	0.7950	−1.78	0.074	−0.9786
	Awareness	−0.5902	0.2204	−2.68	0.007	−0.4153
	Knowledge	−0.8844	0.5270	−1.68	0.093	−0.5320
	Total	−0.0007	0.1226	−0.01	0.995	−0.0004
	Gambling	0.3067	0.2342	1.31	0.19	0.3014
Indirect effects: structural						
GD severity	Emotion dysregulation	0.0067	0.0043	1.57	0.117	0.0480
	Impulsivity	0.0046	0.0033	1.42	0.156	0.0335
	Age	−0.0107	0.0062	−1.74	0.082	−0.0435
	Sex (male)	−0.1688	0.2964	−0.57	0.569	−0.0133
	Social index (lower)	0.1239	0.1004	1.23	0.217	0.0390
Psychopathology	Emotion dysregulation	0.0143	0.0148	0.97	0.333	0.0154
	Impulsivity	0.0099	0.0107	0.93	0.353	0.0107
	Age	−0.1002	0.0692	−1.45	0.147	−0.0608
	Sex (male)	−2.7938	3.5091	−0.80	0.426	−0.0329
	Social index (lower)	2.4033	0.9206	2.61	0.009	0.1130
Advertising	Age	−0.0017	0.0016	−1.07	0.286	−0.0341
	Sex (male)	−0.0490	0.0673	−0.73	0.466	−0.0192
	Social index (lower)	0.0434	0.0301	1.44	0.149	0.0680
Indirect effects: measurement						
Involvement	Emotion dysregulation	−0.0095	0.0029	−3.27	0.001	−0.2337
	Impulsivity	−0.0066	0.0028	−2.35	0.019	−0.1631
	Age	0.0024	0.0018	1.33	0.182	0.0333
	Sex (male)	0.0696	0.0871	0.80	0.424	0.0188
	Social index (lower)	−0.0072	0.0632	−0.11	0.909	−0.0078
Awareness	Emotion dysregulation	−0.0039	0.0020	−1.97	0.048	−0.0992
	Impulsivity	−0.0027	0.0016	−1.69	0.092	−0.0692
	Age	0.0010	0.0009	1.17	0.242	0.0142
	Sex (male)	0.0289	0.0381	0.76	0.447	0.0080
	Social index (lower)	−0.0030	0.0260	−0.12	0.908	−0.0033
Knowledge	Emotion dysregulation	−0.0059	0.0021	−2.77	0.006	−0.1270
	Impulsivity	−0.0041	0.0019	−2.11	0.035	−0.0886
	Age	0.0015	0.0012	1.29	0.198	0.0181
	Sex (male)	0.0434	0.0549	0.79	0.43	0.0102
	Social index (lower)	−0.0045	0.0395	−0.11	0.909	−0.0042
Total	Emotion dysregulation	0.0000	0.0008	−0.01	0.995	−0.0001
	Impulsivity	0.0000	0.0006	−0.01	0.995	−0.0001
	Age	0.0000	0.0002	0.01	0.995	0.0000
	Sex (male)	0.0000	0.0060	0.01	0.995	0.0000
	Social index (lower)	0.0000	0.0006	−0.01	0.995	0.0000
Gambling	Emotion dysregulation	0.0021	0.0011	1.88	0.059	0.0720
	Impulsivity	0.0014	0.0009	1.57	0.115	0.0502
	Age	−0.0005	0.0005	−1.14	0.256	−0.0103
	Sex (male)	−0.0150	0.0200	−0.75	0.451	−0.0058
	Social index (lower)	0.0016	0.0137	0.11	0.909	0.0024
Total effects: structural						
Impulsivity	Age	−0.2688	0.1220	−2.20	0.028	−0.1506
	Sex (male)	−1.6326	6.2950	−0.26	0.795	−0.0177
	Social index (lower)	1.9968	1.5689	1.27	0.203	0.0866
GD severity	Emotion dysregulation	0.0179	0.0104	1.71	0.087	0.1285
	Impulsivity	0.0354	0.0102	3.47	0.001	0.2570
	Advertising	1.0000	(constrd)			0.2012
	Age	−0.0054	0.0167	−0.32	0.747	−0.0218
	Sex (male)	−0.7842	0.8590	−0.91	0.361	−0.0619
	Social index (lower)	−0.3131	0.2180	−1.44	0.151	−0.0986
Psychopathology	Emotion dysregulation	0.3764	0.0594	6.33	0	0.4041
	Impulsivity	0.2796	0.0580	4.82	0	0.3031
	Advertising	2.1435	1.7837	1.20	0.229	0.0644
	Age	0.0820	0.1133	0.72	0.469	0.0498
	Sex (male)	−3.3138	5.8418	−0.57	0.571	−0.0390
	Social index (lower)	2.1183	1.4584	1.45	0.146	0.0996
Advertising	Emotion dysregulation	0.0067	0.0043	1.57	0.117	0.2388
	Impulsivity	0.0046	0.0033	1.42	0.156	0.1666
	Age	−0.0017	0.0016	−1.07	0.286	−0.0341
	Sex (male)	−0.0490	0.0673	−0.73	0.466	−0.0192
	Social index (lower)	0.0051	0.0440	0.12	0.908	0.0080
Emotion dysregulation	Age	−0.0666	0.1190	−0.56	0.576	−0.0376
	Sex (male)	−6.2096	6.1437	−1.01	0.312	−0.0682
	Social index (lower)	5.1201	1.5312	3.34	0.001	0.2244
Total effects: measurement						
Involvement	Emotion dysregulation	−0.0095	0.0029	−3.27	0.001	−0.2337
	Impulsivity	−0.0066	0.0028	−2.35	0.019	−0.1631
	Advertising	−1.4184	0.7950	−1.78	0.074	−0.9786
	Age	0.0024	0.0018	1.33	0.182	0.0333
	Sex (male)	0.0696	0.0871	0.80	0.424	0.0188
	Social index (lower)	−0.0072	0.0632	−0.11	0.909	−0.0078
Awareness	Emotion dysregulation	−0.0039	0.0020	−1.97	0.048	−0.0992
	Impulsivity	−0.0027	0.0016	−1.69	0.092	−0.0692
	Advertising	−0.5902	0.2204	−2.68	0.007	−0.4153
	Age	0.0010	0.0009	1.17	0.242	0.0142
	Sex (male)	0.0289	0.0381	0.76	0.447	0.0080
	Social index (lower)	−0.0030	0.0260	−0.12	0.908	−0.0033
Knowledge	Emotion dysregulation	−0.0059	0.0021	−2.77	0.006	−0.1270
	Impulsivity	−0.0041	0.0019	−2.11	0.035	−0.0886
	Advertising	−0.8844	0.5270	−1.68	0.093	−0.5320
	Age	0.0015	0.0012	1.29	0.198	0.0181
	Sex (male)	0.0434	0.0549	0.79	0.43	0.0102
	Social index (lower)	−0.0045	0.0395	−0.11	0.909	−0.0042
Total	Emotion dysregulation	0.0000	0.0008	−0.01	0.995	−0.0001
	Impulsivity	0.0000	0.0006	−0.01	0.995	−0.0001
	Advertising	−0.0007	0.1226	−0.01	0.995	−0.0004
	Age	0.0000	0.0002	0.01	0.995	0.0000
	Sex (male)	0.0000	0.0060	0.01	0.995	0.0000
	Social index (lower)	0.0000	0.0006	−0.01	0.995	0.0000
Gambling	Emotion dysregulation	0.0021	0.0011	1.88	0.059	0.0720
	Impulsivity	0.0014	0.0009	1.57	0.115	0.0502
	Advertising	0.3067	0.2342	1.31	0.19	0.3014
	Age	−0.0005	0.0005	−1.14	0.256	−0.0103
	Sex (male)	−0.0150	0.0200	−0.75	0.451	−0.0058
	Social index (lower)	0.0016	0.0137	0.11	0.909	0.0024

Emotion dysregulation measured as the DERS total. Impulsivity measured as the UPPS-P total. GD severity measured as the SOGS total. Psychopathology measured as the SCL-90R PST. Sample size: *n* = 210.

The latent variable assessing advertising impact achieved significant coefficients for all the indicator variables used in its definition, except for the HANSS: total. The latent variable was higher when: (i) involvement was lower (HANSS); (ii) awareness was lower (HANSS); (iii) knowledge was lower (HANSS) or higher (GASKI). Higher values on this latent measure (‘Advertising’) suggested participants were more susceptible to the effects of gambling advertising.

The path analysis showed that higher gambling severity level was directly associated with higher perceived impact by gambling advertising, lower social index positions, and higher impulsivity levels. In addition, gambling advertising was a mediator in the paths between (i) emotion regulation and gambling severity, and (ii) impulsivity and gambling severity. Regarding the global psychopathology distress, higher SCL-90-R PST scores were related to more difficulties in the emotion regulation, higher impulsivity, and older age. Moreover, higher SOGS score (GD severity) was associated with SCL-90-R PST (higher number of psychological comorbid problems). These results are not discussed as they are not directly associated to the aim of the study regarding gambling advertising effects.

## Discussion

The present paper explored the relationship between gambling advertising and other gambling-related variables to determine its role (if any) in increasing gambling severity. Gambling advertising was operationalized as a latent variable comprising three measures: attitudes towards gambling advertising, perceived knowledge about how persuasion works in advertising, and perceived impact of gambling advertising. The results indicated that two of these three measures related to gambling advertising were meaningful predictors of gambling severity. First, the higher the perceived impact of gambling advertising (meaning lower scores), the higher the susceptibility of participants to gambling advertising. This was true for all the components of the scale: knowledge, awareness, and involvement (Hanss et al., 2015). Second, the higher the scores on attitude (i.e., more positive attitudes towards gambling advertising) (Gaski and Etzel, 1986), the higher the susceptibility to gambling advertising. The third measure (Bearden et al., 2001) was not found to be meaningful. Altogether, the constructed latent variable of gambling advertising had a significant effect on gambling severity among participants.

The results support the existing consensus that gamblers who perceive gambling advertising to have negative effects on their mental health and/or gambling behaviour are those experiencing more gambling problems (Binde, 2009; Hanss et al., 2015; Syvertsen et al., 2022; McGrane et al., 2023). The present study consolidates the existing evidence in one meaningful way, by demonstrating the association between gambling advertising impact and problem gambling among gamblers who had their GD diagnosis following interviews with a mental health professional. Additionally, the results on the attitudinal component also aligned well with previous studies in which gamblers with GD showed more positive attitudes towards gambling advertising (Derevensky et al., 2010; Hing et al., 2015b), perhaps because they normalize gambling advertising due to their constant exposure to it.

A major finding of the study was the mediating role of gambling advertising in the relationship of emotion regulation and impulsivity with gambling severity. Both emotion regulation and impulsivity have been consistently associated with problem gambling, with gamblers more emotionally dysregulated and impulsive having greater gambling severity (Nower and Blaszczynski, 2006; Estévez et al., 2017; Savvidou et al., 2017; Mestre-Bach et al., 2020). However, such associations had not been previously explored to test whether gambling advertising mediated the relationships. Gamblers scoring higher on emotion dysregulation can be more susceptible to stimuli that remind them of strategies to regulate themselves, even if these strategies are maladaptive as it is the case of problem gambling. An emotionally dysregulated gambler is more likely to resort to gambling to compensate for/neutralize uncomfortable emotional states. Therefore, exposure to gambling advertising to gamblers in moments of high emotional dysregulation is a public health concern that calls for regulatory actions.

Similarly, the mediation of impulsivity and gambling severity by gambling advertising calls for identical actions. Impulsive gamblers are more likely to respond positively to promotions that present themselves as financially beneficial without analyzing the true cost involved in accepting them (Newall et al., 2019). In many gambling contexts such as in-play sports betting, gamblers are watching live content that creates gambling opportunities that must be seized within seconds or otherwise be missed (Russell et al., 2018; Lopez-Gonzalez et al., 2020a). This type of betting is impulsive by definition because of the short span of time between real-world events and gambling behaviour. Many sport and horse racing events that produce in-play betting markets have huge audiences and are consequently large platforms for advertisers (gambling operators and others). Gamblers with higher impulsivity who also report a greater perceived impact from gambling advertising will be more vulnerable to gambling adverts seen during sport broadcasts because such adverts will entice them to bet on the spur of the moment. In-play betting has been a matter of concern for regulators for a long time (Gambling Commission, 2016; Directorate General for the regulation of gambling [DGOJ], 2017; Conn and Davies, 2020) but the present study puts further pressure on regulators and the gambling industry to minimize the effects of gambling advertising strategies that connect impulsive behaviour and gambling severity.

Furthermore, the study findings provide solid evidence to implement greater regulatory measures on gambling provision by gambling operators. The self-perceived impact of gambling advertising on the attitudes and behaviours of those experiencing GD does not demonstrate per se that gambling advertising caused or promoted the GD, but it is arguably enough to enforce stronger measures that effectively prevent gambling stimuli from reaching them. This is because, as reported by them, the gambling stimuli is distressing, regardless of its association (or not) with GD development or maintenance. Specifically, the practical implications of the present study could be materialized in various forms. Media corporations and big tech companies should commit greater resources to reassuring that individuals with gambling problems are not exposed to any gambling stimuli. In the case of social media, this should be feasible by adjusting profile preferences of users. Similarly, the indirect association of impulsivity and gambling severity via gambling advertising could be practically addressed in online gambling settings by implementing behavioural tracking systems that flag impulsive behaviour indicators (e.g., repeated bets on events of short duration). When such behaviours are detected, promotions that stimulate the continuation of gambling should be ceased.

The present study has some limitations. First, the recruitment of participants was interrupted due to the COVID-19 pandemic. One-third of the sample joined the study after the country’s confinement period, while the others had been assessed prior to the pandemic. The confinement might have affected the exposure to gambling advertising because participants might have stayed at home more frequently, increasing their overall screen time. Second, the data primarily depended on self-reported factors, such as individuals’ perceptions of the influence of gambling advertisements, making it susceptible to specific biases (e.g., recall bias, where participants may have underestimated their exposure to gambling advertising). Social desirability could have influenced responses in two ways: some may have exaggerated the impact of gambling advertising to appear critical and aware, while others may have downplayed it to seem resistant to commercial messages. Furthermore, despite the data spanning 18 months, the study was cross-sectional and did not assess the consistency of the scores over time. In this regard, the path analysis model was employed exploratorily and not as a confirmatory model. However, it should be taken into account that either longitudinal and cross-sectional data can be used when conducting SEM, with adequate models specification in both cases. Future research could use path analysis in longitudinal designs to assess the directionality of the variables involved. Alternatively, experimental studies could be helpful in isolating the specific impact of gambling advertising on gambling severity. Finally, the path analysis proposed a mechanistic model of the influence of gambling advertising impact on gambling severity. This model presupposes two things. First, gambling advertising impact happens as a result of a previous exposure to gambling advertising. And second, the response to gambling advertising is susceptible to the intensity and duration of the expositions (dose˗response relationship). Unfortunately, the present study did not control for gambling exposure.

## Conclusion

Gambling advertising is one of the primary vehicles of the gambling industry to recruit new and maintain existing customers. The present study adds further evidence supporting the notion that gamblers experiencing problems perceive gambling advertising to have a greater impact on them. Gambling advertising was also found to play a mediating role between both emotion regulation and impulsivity with gambling severity. The results can be used to implement restrictions on how frequent gamblers get exposed to gambling adverts, limiting the ability of the gambling operators to target vulnerable gamblers in their advertising campaigns. Furthermore, on gambling advertising messages, featuring structural characteristics of gambling that promote impulsive gambling (e.g., microbets) could be discouraged based on their effect on gamblers with GD.

## Data Availability

The raw data supporting the conclusions of this article will be made available by the authors, without undue reservation.
