# Supplementary material for: The impact of gambling advertising on gambling severity: a path analysis of factors of psychological distress in individuals with gambling disorder
Source: Front Psychol. 2025 Mar 14;16:1523906. doi: 10.3389/fpsyg.2025.1523906 (PMC11949876; doi:10.3389/fpsyg.2025.1523906)
Supplement: Supplementary file 1 [file Table_1.docx]

**Table 1 (Supplementary Material)**

***Table S1 (supplementary)*** Descriptive for the sample *(n=210)*

|  | *n* | *%* |  | *Age-onset-duration* | *Mean* | *SD* |
| --- | --- | --- | --- | --- | --- | --- |
| Sex Female | 15 | 7.1% |  | Chronological age (yrs) | 39.40 | 13.30 |
| Male | 195 | 92.9% |  | Onset of GD (yrs) | 28.87 | 11.91 |
| Marital status Single | 113 | 53.8% |  | Duration GD (yrs) | 5.21 | 5.65 |
| Married | 67 | 31.9% |  | *GD symptom severity* | *Mean* | *SD* |
| Divorced | 30 | 14.3% |  | SOGS total | 10.92 | 3.27 |
| Education Primary | 95 | 45.2% |  | *Impulsivity and emotion regulation* | *Mean* | *SD* |
| Secondary | 88 | 41.9% |  | UPPS-P Total score | 138.62 | 23.75 |
| University | 27 | 12.9% |  | DERS Total score | 92.28 | 23.52 |
| Employed No | 76 | 36.2% |  | *Psychopathology* | *Mean* | *SD* |
| Yes | 134 | 63.8% |  | SCL-90R PST | 49.26 | 21.90 |
| Social index High | 4 | 1.9% |  | *Impact advertising* | *Mean* | *SD* |
| Mean to high | 22 | 10.5% |  | Hanss et al.: Involvement | 2.85 | 0.95 |
| Mean | 22 | 10.5% |  | Hanss et al.: Awareness | 2.60 | 0.94 |
| Mean to low | 86 | 41.0% |  | Hanss et al.: Knowledge | 2.61 | 1.09 |
| Low | 76 | 36.2% |  | Hanss et al.: Total | 2.74 | 0.80 |
| GD type Non strategic | 81 | 38.6% |  | Bearden et al.: Total | 2.99 | 1.18 |
| Strategic | 76 | 36.2% |  | Gaski and Etzel: Gambling | 1.69 | 0.67 |
| Mixed | 53 | 25.2% |  |  |  |  |
| GD modality Offline | 153 | 72.9% |  |  |  |  |
| Online | 44 | 21.0% |  |  |  |  |
| Mixed | 13 | 6.2% |  |  |  |  |

*Note.* GD: gambling disorder. SD: standard deviation.
